# Organic Iodine Improves the Growth Performance and Gut Health of Fujian Yellow Rabbits

**DOI:** 10.3390/ani14131935

**Published:** 2024-06-29

**Authors:** Qinghua Liu, Yeqiu Zhang, Jie Zhang, Zhijian Du, Bixian He, Juanqing Qin, Liping Zhang, Jing Zhang

**Affiliations:** College of Animal Sciences, Fujian Agriculture and Forestry University, Fuzhou 350002, China; liuqh@fafu.edu.cn (Q.L.); 17359760297@163.com (Y.Z.); zj1532571033@163.com (J.Z.); 17607848230@163.com (Z.D.); 13316274137@163.com (B.H.); q1777610@163.com (J.Q.); zlping2024@163.com (L.Z.)

**Keywords:** organic iodine, Fujian yellow rabbits, growth performance, gut health

## Abstract

**Simple Summary:**

Iodine regulates the metabolic pathways of carbohydrates, proteins, fats, minerals, and vitamins in animals. Organic iodine overcomes the problems of low absorption and utilization rate and excessive dosage of traditional inorganic salts. Fujian yellow rabbits, a local elite variety, have a medicinal function, a high reproduction rate, and great carcass quality, presenting a huge market potential. Fujian yellow rabbits are hindgut fermenters, which are typically in a precarious balance. Gut health directly affects the health of the animals, which in turn affects production performance. This study described the effect of organic iodine in Fujian yellow rabbit diets and indicated that organic iodine improves growth performance and increases serum T3 and serum T4 concentrations. Organic iodine ameliorates gut health through adequate intestinal morphology, and active caecum fermentation, and enhances enzyme activity and intestinal immunity when adding 0.5 mg/kg organic iodine in Fujian yellow rabbit diets.

**Abstract:**

Organic iodine is a new trace element additive that is highly efficient in regulating cell growth, function, and metabolism. This study demonstrated that organic iodine improves the growth performance and gut health of Fujian yellow rabbits. A total of 160 healthy rabbits of similar weight were randomly divided into four groups, which were treated with organic iodine (0, 0.5, 1.0, and 1.5 mg/kg) for 60 days. Our results indicated that organic iodine improved the growth performance, including significantly increased BW, ADG, and ADFI, and decreased F/G notably. Organic iodine improved the content of T3, T4, IgM, IgA, and IgM in serum, and intestinal mucosal immunity (IL-1α, IL-2, and sIgA). Organic iodine supplementation ameliorated gut morphometry and morphology, such as higher villus height and lower crypt depth. Organic iodine increased the amount of goblet cells significantly. The 0.5 mg/kg organic iodine most increased the activities of amylase, cellulase, and trypsin in caecum. Organic iodine induced more active caecum fermentation, higher NH_3_-N, acetic acid, propionic acid, and butyric acid, while lowering PH. In conclusion, organic iodine improved the growth performance and gut morphometry and morphology, and increased caecum enzyme activities, active caecum fermentation, and intestinal immunity of Fujian yellow rabbits.

## 1. Introduction

Iodine is a trace element necessary for the animal body to maintain normal life activities. It enhances enzyme activity, regulates protein and fat metabolism, and also releases iodine into the blood and then into the thyroid gland to be utilized to synthesize thyroxine [1]. Moreover, iodine deficiency causes thyroid gland enlargement and indirectly influences the growth rate [2,3]. Organic iodine is a new type of trace element and refers to iodine combined with organic matter, which is safe, stable, and highly utilized [4]. Fujian yellow rabbit is a unique meat rabbit in Fujian Province. It has the characteristics of wide adaptability, high reproduction rate, high survival rate of young rabbits, and high slaughter rate of meat rabbits. Fujian yellow rabbits are monogastric herbivores and hindgut fermenters that rely on cecotrophy to ensure maximum nutrient absorption from their diet [5]. The intestinal health of yellow rabbits directly affects their production performance. Studies have shown that iodine addition is beneficial for rumen fermentation and forage degradation [6], improving milk production [7], improving reproductive performance [8], improving production performance, reducing feed-to-weight ratio [9], improving antioxidant capacity [10], and improving immunity [11]. According to the nutritional requirements of meat rabbits in China (NY/T4049-2021) [12] and the feeding standards of meat rabbits in France (1993), the iodine requirement of growing rabbits is 1 mg∙kg^−1^. Therefore, in this study, organic iodine was used as the material and weaned Fujian yellow rabbits were used as the test animals to carry out a fattening trial of organic iodine fed to Fujian yellow rabbits to explore the appropriate additive amount of organic iodine and provide a reference for its scientific use in meat rabbit production.

## 2. Materials and Methods

### 2.1. Experimental Materials

Fujian yellow rabbits were supplied by Fujian Chunlong Agriculture and Animal Husbandry Technology Co., Ltd. (Fuzhou, China), and organic iodine was supplied by Sichuan Jilongda Biotechnology Group Co., Ltd. (Deyang, China). The main raw material is organic chelating iodine (LOT NO: 2003241078).

### 2.2. Experimental Methods

The rabbit house is fully cleaned and disinfected by the single cage feeding method. Feed once a day at 8:00 and 18:00 and also provide ad libitum access to drinking water. Keep the rabbit house quiet, clean, tidy, and disinfect every 7 days. Observe and record the production status of rabbits, and do a good job in the isolation and treatment of abnormal rabbits. The experimental feed design adheres strictly to the French (1993) feeding standards, and sufficient iodine for growth and development is added to the diet to meet the normal physiological needs of rabbits. The regular feeding period begins with weighing on a fasting stomach before the morning feeding on the first day, which is the initial weight of the test rabbit. On the 53rd day, the rabbit was weighed before morning feeding, which was the final weight of the test rabbit. The total weight gain and daily weight gain were calculated. During the regular feeding period, the daily feed intake was recorded. The remaining feed in the trough was collected, weighed, and recorded, and the average daily feed intake (ADFI), average daily gain (ADG), and feed-to-gain ratio (F/G) were calculated.

### 2.3. Experimental Design

#### 2.3.1. Animal Experiments

A total of 160 healthy rabbits of similar weights (0.79 ± 0.01 kg) were treated with organic iodine (0, 0.5, 1.0, 1.5 mg/kg). All rabbits were fed their corresponding diet for 60 consecutive days (preparatory feeding for 7 days). All the rabbits were fed the basal diet and were maintained according to the French rabbit feeding standard formulation (1993) (Table 1). The Ethical and Animal Welfare Committee of Fujian Agriculture and Forestry University approved this study. Animal care and treatment were in strict accordance with the standards of the Experimental Animals Care and Use Guide of the Fujian Agriculture and Forestry University.

#### 2.3.2. Sample Collection

Rabbits were fasted for 12 h and then sacrificed. The tissues were immediately separated, frozen in liquid nitrogen, and stored in a cryogenic refrigerator at −80 °C. The remaining samples were stored at −20 °C. The blood was centrifuged for 15 min at 3000× *g* at 4 °C, and the serum samples were stored at −20 °C.

#### 2.3.3. Growth Performance

The body weight (BW) of Fujian yellow rabbits was recorded on days 0 and 53. The average daily gain (ADG), average daily feed intake (ADFI), and feed/gain (F/G) of rabbits in each experimental group were calculated.

#### 2.3.4. Serum Biochemical Analysis

In serum, we obtained the triiodothyronine (T3), thyroxine (T4), immunoglobulin A (IgA), immunoglobulin (IgG), and immunoglobulin M (IgM) according to the instructions of the commercial diagnostics kits (Nanjing Jiancheng Biotechnology Co., Ltd., Nanjing, China).

#### 2.3.5. Intestinal Mucosal Immunity

IL-1α, IL-2, and sIgA in the duodena, jejunum, and ileum of rabbits were determined by ELISA commercial diagnostics kits (Shanghai Preferred Biotechnology Co., Ltd., Shanghai, China).

#### 2.3.6. Intestinal Enzyme Activity

Amylase, cellulase, and trypsin in cecal contents of rabbits were determined according to the instructions of the commercial diagnostics kits (Nanjing Jiancheng Biotechnology Co., Ltd., Nanjing, China).

#### 2.3.7. Gut Morphometry and Morphology

The gut samples of rabbits were fixed in 4% paraformaldehyde after saline flushes. Fixed samples were dehydrated through graded alcohols hyalinized by xylene, and then embedded in paraffin blocks. Stained kidney sections with hematoxylin–eosin (H&E) were examined at 5 μm for 10 photos. The sections were observed under an Eclipse Ci-L camera microscope. The villus height (VH), depth of recess (CD), and goblet cell (GC) of five intact intestinal villi in each section were measured, and the villus height/crypt depth (V/C) was calculated and recorded.

#### 2.3.8. Cecal Fermentation

The pH values of the upper, middle, and lower parts of the cecum were measured using a pH meter (PHSJ-3F, Leici, Shanghai, China). The content of NH3-N was determined by the phenol-sodium hypochlorite colorimetric method [13]. The contents of acetic acid, propionic acid, and butyric acid were determined by gas chromatography (Agilent 7890N, Santa Clara, CA, USA) with Agilent DB-23 chromatographic column (60 m × 0.25 mm × 0.25 μm), and nitrogen as the carrier gas. The inlet temperature was set at 270 °C and the detector was set at 280 °C. The flow rate of hydrogen, nitrogen, and air are, respectively, set to 50 mL·min^−1^, 30 mL·min^−1^, and 500 mL·min^−1^. The split ratio is set to 50: 1; the initial temperature was maintained at 80 °C for 4 min, and then increased to 180 °C at 10 °C min^−1^ for 1 min, and the flow rate was set to 2 m·min^−1^. The injection volume was 2 μL.

#### 2.3.9. Cecal Flora Structure

Cecal content was collected into 5 mL sterilized vials that were promptly refrigerated and frozen until DNA extraction. Genomic DNA was extracted using the cetyl trimethylammonium bromide lysis buffer method (CTAB) [14]. The sample DNA was placed in a centrifuge tube and diluted to 1 ng·μL^−1^ with sterile water. According to the selection of sequencing region, the V3–V4 region of 16S rRNA genes was evaluated using specific primers with Barcode by adopting diluted genomic DNA as a template. We applied Qubit and Q-PCR for quantitative measurement before library preparation following the instructions for the TruSeq DNA PCR-Free Sample Preparation Kit (Illumina, Inc., San Diego, CA, USA). The Illumina HiSeq 2500 platform (Illumina, Inc., San Diego) was employed to sequence with the Paired-End method.

The forecast of cecal flora structure functional pathways included the clustering of OTUs, the taxonomic analysis of PCoA and LEfSe, the employment of PICRUSt (2.3.0) software, and the comparison of 16S rDNA sequencing information with Gene Annotation Database KEGG (Kyoto Encyclopedia of Genes and Genomes, Archive) for efficacy data.

### 2.4. Statistical Analysis

Results were presented as the mean standard deviation, where *p* < 0.05 indicates a significant difference, and *p* < 0.01 indicates an extremely significant difference. Duncan multiple range tests were employed to compare means after one-way ANOVA was carried out using an SPSS software package (version 25 for Windows; SPSS Inc., Chicago, IL, USA).

## 3. Results

### 3.1. Growth Performance

Growth performance parameters are shown in Table 2. Organic iodine of 1.5 mg∙kg^−1^ improved the growth performance, including final weight significantly increasing by 14.36% (*p* < 0.01), ADG increasing by 23.58% (*p* < 0.01), ADFI increasing by 4.48% (*p* < 0.01), and F/G decreasing by 15.38% (*p* < 0.01). Organic iodine improves growth performance.

### 3.2. Serum Biochemical Indicators

Data on serum biochemical indicators are shown in Table 3. Organic iodine in-creased T3 (*p* < 0.01) and T4 (*p* < 0.01) concentrations in blood serum. Organic iodine improved the serum immunoglobulin, including IgA significantly increasing by 44.21% (*p* < 0.05), and IgA and IgM increasing significantly (*p* < 0.05).

### 3.3. Intestinal Mucosal Immunity

The data on intestinal mucosal immunity are shown in Table 4. Organic iodine improved intestinal mucosal immunity, including IL-1α, IL-2, and sIgA. The levels of interleukin IL-1α, IL-2, and SIgA in the intestinal mucosa of group I were highly significantly higher than the control group (*p* < 0.01). The levels of IL-1α, IL-2, and SIgA were significantly higher than the control group (*p* < 0.05) in the intestinal mucosa of group II.

### 3.4. Intestinal Histomorphology

**Figure 1:**
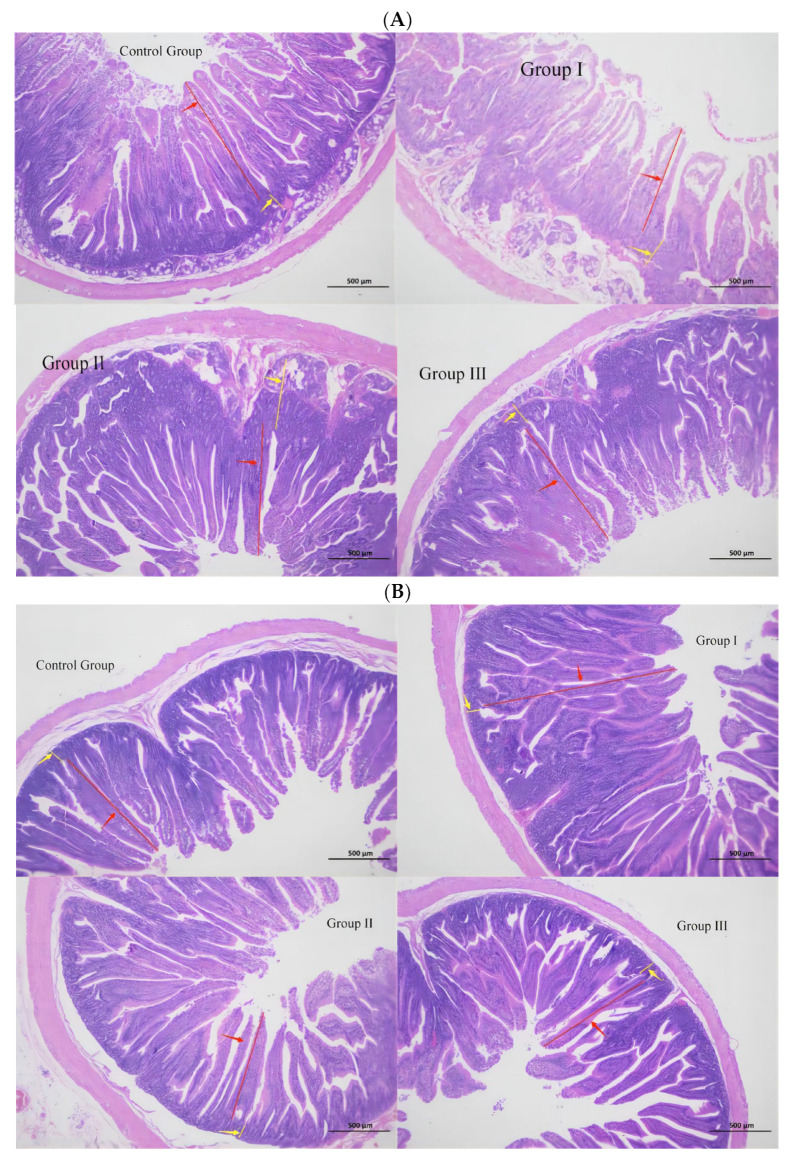

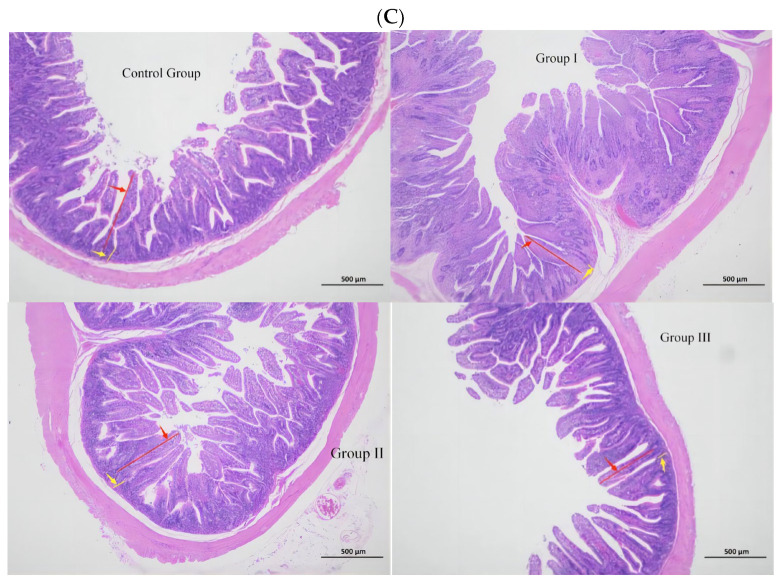
Effect of organic iodine on the intestinal morphology of Fujian yellow rabbits. (**A**): duodenum, (**B**): jejunum, (**C**): ileum. (H&E, 40×).

A hematoxylin–eosin staining was performed to observe intestinal morphology (Figure 1 and Table 5). The villus height and crypt depth of the duodenum in group I were significantly higher than those in group II (*p* < 0.05). The number of goblet cells in group II was the highest and 2.66 times higher than that in the control group (*p* < 0.01); duodenum, jejunum, and ileum in group III were 1.42 times (*p* < 0.05), 1.24 times (*p* > 0.05), and 1.82 times (*p* < 0.01) higher than those in the control group.

### 3.5. Intestinal Enzyme Activity

As shown in Table 6, the activities of amylase, cellulase, and trypsin in group I were the highest, and the cellulase activities were significantly higher than those in the control group (*p* < 0.05). Organic iodine of 0.5 mg·kg^−1^ most increased the activities of amylase, cellulase, and trypsin in the caecum.

### 3.6. Cecal Fermentation

As shown in Table 7, organic iodine induced more active caecum fermentation, and higher NH_3_-N, acetic acid, propionic acid, and butyric acid, while lowering PH. Compared with the control group, pH and ammonia nitrogen in group I increased by 5.48% (*p* < 0.05) and 57.19% (*p* < 0.05), and acetic acid content in group II increased by 22.55% (*p* < 0.05), respectively. Group III had the highest pH value, which was significantly higher than that of group I (*p* < 0.01). Group II had the highest propionic acid content, which was significantly higher than that of group III (*p* < 0.05), but there was no significant difference compared to the control and group I (*p* > 0.05).

### 3.7. Cecal Flora Structure

#### 3.7.1. Sequencing Data and OTU Clustering

**Figure 2:**
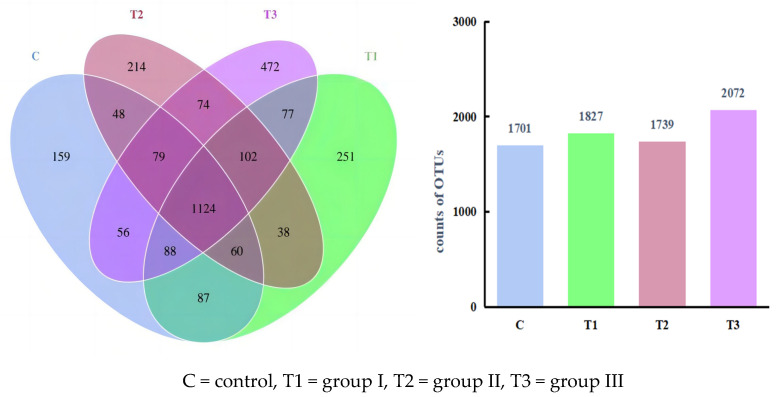
The OTU Venn diagram of microflora in the cecum of Fujian yellow rabbits.

Based on the Illumina Nova sequencing platform, the Effective Tags of the sequenced samples were clustered into OTUs with 97% consistency, yielding Figure 2. No significant differences were observed in the four groups.

#### 3.7.2. Alpha and Beta Diversity Analysis

Table 8 demonstrates the comparative results of the alpha diversity indices; the coverage rate of each group was more than 99%, indicating that the libraries constructed in this experiment could reflect the bacterial diversity. The Ace index, Chao1 index, Shannon index, and Simpson index of each group were not significantly different (*p* > 0.05), indicating that there was no significant difference in the composition of microbial community structure.

**Figure 3:**
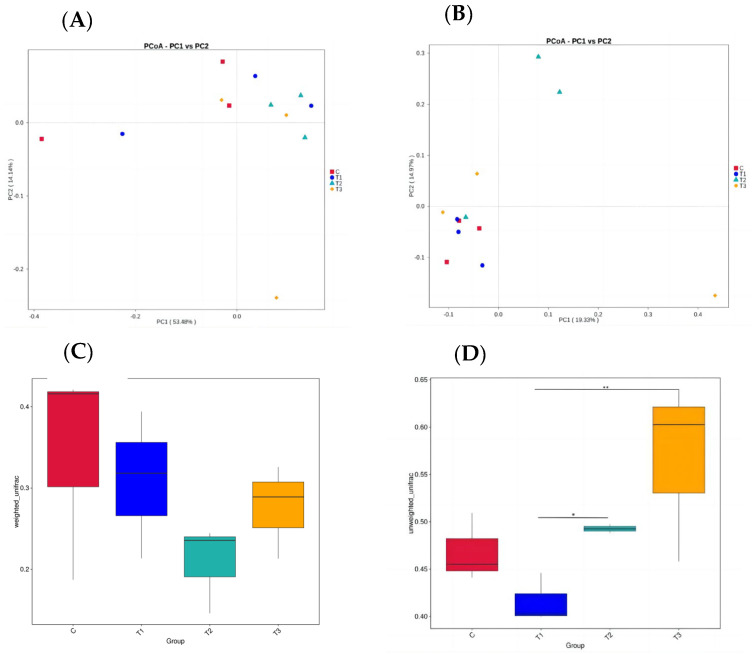
Beta diversity analysis of microflora in the cecum of Fujian yellow rabbits. (**A**): PCoA of rumen microbial composition based on Unweighted Unifrac. (**B**): PCoA of rumen microbial composition based on Unweighted Unifrac. (**C**): Unweighted Unifrac analysis box diagram. (**D**): Weighted Unifrac analysis box diagram.

As illustrated in Figure 3, PCoA analysis showed that the distribution of the four groups was relatively dispersed. As shown in Figure 3D, compared with the beta diversity of group I, group II was significantly higher (*p* < 0.05), and group III was highly significant (*p* < 0.01).

#### 3.7.3. Relative Abundance Analysis at the Phylum, Family, and Genus Level

**Figure 4:**
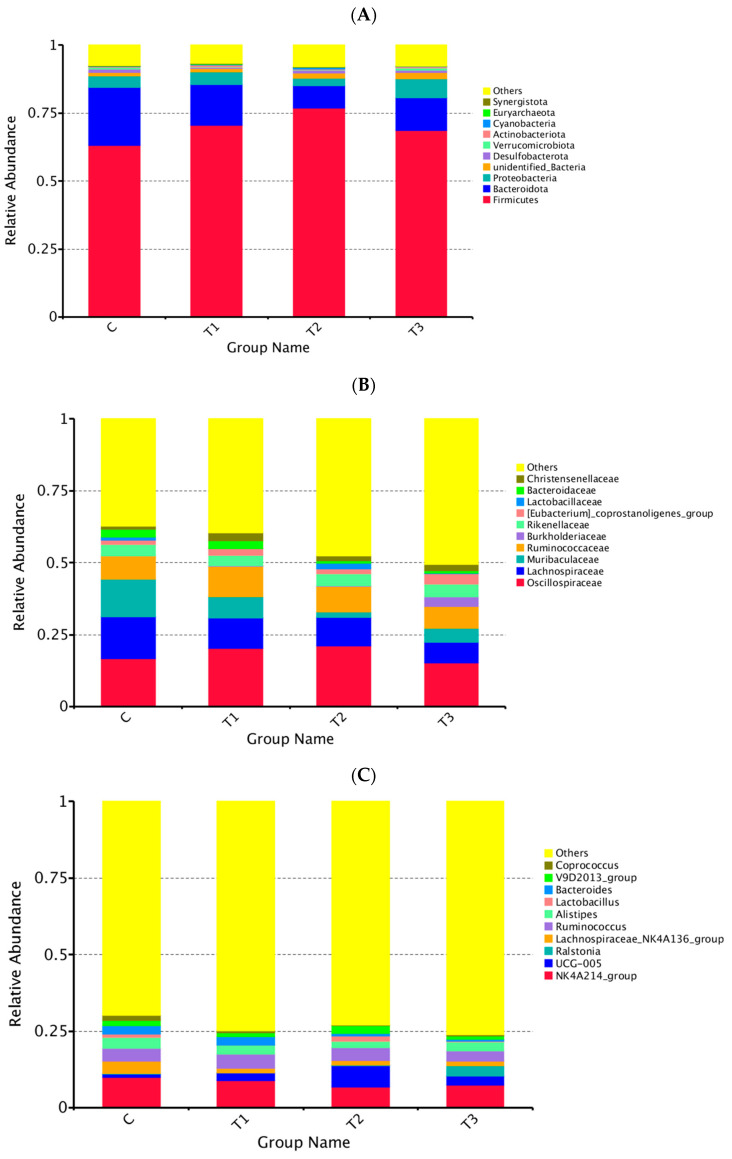
Relative abundance of OTUs about the cecal microflora of Fujian yellow rabbits on different levels. (**A**): phylum; (**B**): genus; (**C**): family.

As shown in Figure 4, at the phylum level, the groups were dominated by the Firmicutes, Bacteroidetes, and Proteobacteri; organic iodine was able to increase the relative abundance of the Firmicutes and decrease the relative abundance of the Bacteroidetes (*p* > 0.05).

At the family level, the dominant species in each group were Oscillospiraceae, Lachnospiraceae, Muribaculaceae, and Ruminococcaceae. The relative abundance of Muribaculaceae in the control group was higher than that in the experimental group and significantly higher than that in group II (*p* < 0.05), while the relative abundance of the remaining groups did not differ significantly (*p* > 0.05).

At the genus level, the dominant species of cecal flora in each group were NK4A214_group, UCG-005, Ralstonia, Lachnospiraceae_NK4A136_group, Ruminococcus, and Alistipes, but there was no significant difference between the groups (*p* > 0.05).

#### 3.7.4. Intergroup Differential Species and Functional Prediction

**Figure 5:**
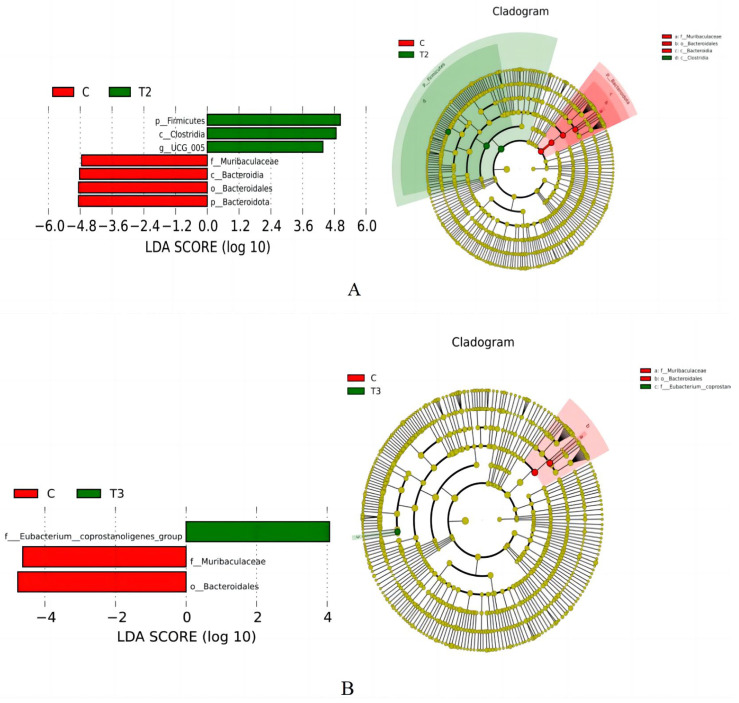
LDA value distribution and evolutionary branching of cecal microflora in Fujian yellow rabbits. (**A**) LEfSe analysis of control group and group II (**B**) LEfSe analysis of control group and group III.

Figure 5 demonstrates the results of LEfSe analysis. As can be seen from Figure 5A, the control group had a higher abundance of Muribaculaceae, Bacteroidia, Bacteroidales, and Bacteroidote, and group II had a higher abundance of Firmicutes, Clostridia, and UCG_005. As shown in Figure 5B, the abundance of Muribaculaceae and Bacteroidales was higher in the control group, and the abundance of Eubacterium_coprostanoligenes_group was higher in Group III.

**Figure 6:**
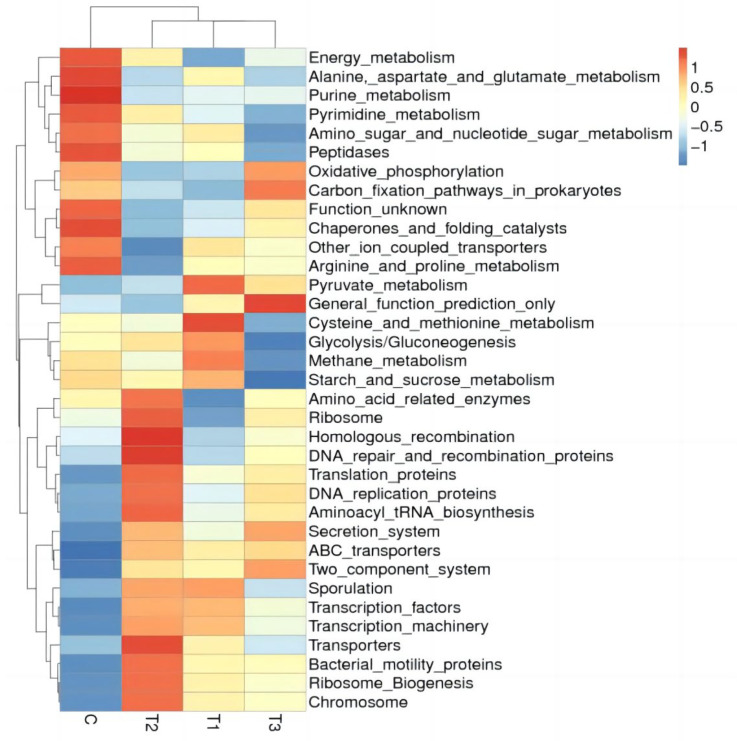
Function annotation cluster heatmap of cecal microflora in Fujian yellow rabbits.

**Figure 7:**
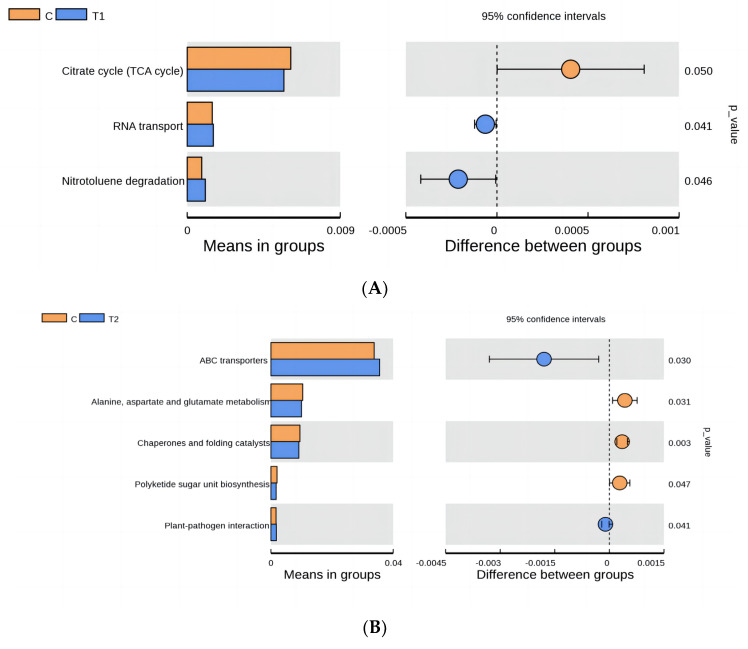

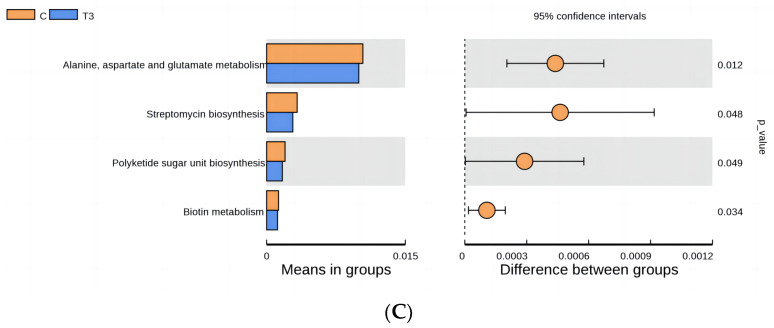
Function prediction and analysis of cecal microflora in Fujian yellow rabbits. (**A**) Control group and group I (**B**) Control group and group II (**C**) Control group and group III.

The functional pathways of cecum flora were predicted by comparison with KEGG (Figure 6). As shown in Figure 7A, TCA cycle function related to nutrient metabolism was enriched in the control group; RNA transporter related to the processing of genetic information and nitrotoluene degradation of nutrient metabolism were enriched in group I.

As shown in Figure 7B, the functions of alanine, aspartate and glutamate metabolism, polyketide sugar unit biosynthesis, and chaperones and folding catalysts related to genetic information processing were enriched in the control group. Group II was enriched for ABC transporters and plant–pathogen interaction functions related to organic systems.

As shown in Figure 7C, the control group was associated with nutrient metabolism with alanine, aspartate, and glutamate metabolism, and streptomycin biosynthesis, polyketide sugar unit biosynthesis, biosynthesis, biotin metabolism being feature-rich.

## 4. Discussion

The indiscriminate abuse of antibiotics can easily lead to the formation of drug-resistant bacteria in the intestines of livestock and poultry, resulting in microbiota disorder over the years. In recent years, antibiotics have been replaced with alternatives like probiotics [15], enzymes [16], and organic acids [17], to improve growth performance, gut health, and organism development and metabolism. Our current study showed that organic iodine significantly improved the growth performance and gut health of Fujian yellow rabbit over the experimental period of 60 days in a dose-dependent manner.

Iodine exerts a physiological function and promotes the growth and development of the body through thyroxine. Adding an appropriate amount of iodine to the diet can improve the growth performance of animals, but the demand varies by age, production, and growth status. Shen et al. [18] showed that the effect of body weight and average feed intake was the best when the iodine addition level was 0.8 mg·kg^−1^ g. Sarlak et al. [19] found that dietary supplementation with ethylenediamine dihydroiodine significantly improved feed intake and egg production performance of layers. Similarly, another study reported that the levels of 0.50 mg·kg^−1^ iodine had respectable effects on growth performance in growing yaks’ diets [3]. Röttger et al. [20] considered that adding potassium iodide to the feed can improve the feed conversion rate of broilers aged 21–35 days. In this experiment, the final weight, average feed intake, and average daily gain of group III were significantly higher than those of the control group, and the feed-to-weight ratio was significantly lower than that of the control group, indicating that the addition of 1.5 mg·kg^−1^ iodine could improve the growth performance of Fujian yellow rabbits, which was consistent with the results of Li et al. [21] and Xu et al. [22].

Iodine is an essential trace element and contributes to thyroid synthesis for all animal species [23]. Also, it plays a role in energy metabolism in animals primarily by binding to T3, T4, and iodotyrosine precursor. T3 is responsible for most of the physiological functions of TH in extrathyroidal tissues, while T4 is the main hormone for thyroid synthesis [24,25,26]. The dynamics of T3 and T4 creation depend on the supply of proper amounts of essential iodine in feeds [27]. Yang Guozhong et al. [28] believed that the content of T3 and T4 in the experimental group was significantly higher than that in the control group when 0.8 mg·kg^−1^ iodine was added to the diet. In the experimental group, it was significantly higher than that in the control group. The results of this experiment were similar to the conclusion of Liu et al. [29]. The contents of T3 and T4 in the experimental group increased first and then decreased. When 0.5 mg·kg^−1^ organic iodine was added, the T3 value was the highest, whereas the T4 value was the highest with the addition of 1.0 mg·kg^−1^. Observed changes in the thyroid hormone concentration as an effect of increased iodine levels may elucidate the activation of the negative feedback regulation mechanism in order to maintain the normal level. The concentration of immunoglobulin in serum can reflect the immune function of the body. Serum IgA, IgG, and IgM are widely involved in the immune response of the body. The higher the concentration, the stronger the immune function of the body [30,31]. Aiming to explore the effect of iodine levels on serum immunoglobulin of New Zealand white rabbits, Liu et al. [29] stated that the content of IgA was higher than that of other groups when the amount of iodine added was 0.5 mg·kg^−1^. Based on our findings, by means of adding 1.5 mg·kg^−1^ of organic iodine, the content of IgA, IgG, and IgM in the serum of Fujian yellow rabbits was significantly higher than that of the control group, and it has a good effect on the immunity of Fujian yellow rabbits.

Iodine plays a beneficial role in gut health by improving gut development, enhancing gut barrier function, and regulating gut microflora.

The intestinal tract is crucial for digestion, absorption, and utilization of nutrients as the main digestive organ of rabbits. Morphological integrity of intestinal mucosa is the premise of exerting digestive function. The mucus secreted by goblet cells is of great significance to maintain the integrity and stability of the intestinal mucosa by forming a protective layer of mucous on the intestinal surface [32,33]. According to previous studies, large intestinal VH value, small CD value, and increased V/C value emphasize that the intestines are in full contact with nutrients, and possess strong absorption for feed conversion [34,35,36]. Behroozlak pointed out that iodine supplementation can reduce the relative weight of the jejunum of broilers [37]. Our outcomes were basically consistent with those of Huang [38] who mentioned that the villi height and villi–crypto ratio of the duodenum, jejunum, and ileum of yellow-feathered broilers supplemented with iodine were significantly higher. Meanwhile, the addition of organic iodine to the diets was proved to improve the morphology of the small intestine. When the addition amount was 1.5 mg·kg^−1^, the small intestine had the best digestion and absorption effect on nutrients.

Digestive enzymes are proteins with special functions. It is an important indicator for evaluating the production performance and digestive capacity of animals. The digestive and absorptive capacity of the gastrointestinal tract depends on digestive enzyme activity. The higher the enzymatic activity, the stronger the digestive and absorptive capacity of the gastrointestinal tract [39,40,41]. Cellulase can catalyze the degradation of cellulose and hemicellulose and improve the utilization rate of crude fiber in animal body [2]. The research of Mehri demonstrated that iodine could enhance enzyme activity [2]. The present study showed a positive influence of the supplemented diets on the digestion and absorption of nutrients, and the promotion of growth and development. After supplementing the rabbits’ diets with 0.5 mg·kg^−1^ organic iodine, the amylase, cellulase, and trypsin activities of the experimental rabbits were the highest, and the cellulase activity was significantly higher than that of the control group.

Integrity of the intestinal barrier is an important guarantee for intestinal health [42]. The knowledge of how the organic iodine affects the immune function of livestock and poultry remains limited at present. In our study, with the supplementation of 0.5 mg·kg^−1^ organic iodine, the contents of IL-1α, IL-2, and SIgA in the intestinal mucosa were significantly higher than those in the control group, indicating that organic iodine could regulate the level of inflammatory factors, promote the secretion of sIgA, and protect the intestinal mucosa. This effect is probably owing to its promotion of the occurrence of inflammatory response to a certain extent, which is conducive to facilitating the removal of pathogens by phagocytes, strengthening the self-immune defense barrier, and playing an immunoregulatory role in Fujian yellow rabbits.

Rabbits are monogastric hindgut fermenters [14]. The cecum is an important fermentation organ. It is strikingly complex and highly developed, and has the ability to decompose indigestible lignin and cellulose through microbial fermentation [43,44]. To our knowledge, there is a lack of relevant research on supplementing organic iodine in the diet of rabbits. The present study revealed that when the level of organic iodine was 0.5 mg·kg^−1^, the pH value was the lowest and the content of NH_3_-N was significantly higher than that of group III and the control group. This represented the suitable acidic environment for the growth and reproduction of cecal flora, and for the microorganisms to decompose proteins effectively. The contents of acetic acid, propionic acid, and butyric acid in the cecum of group I and group II were higher than those in the control group, and the content of acetic acid in group II was significantly higher than the control group, indicating that the amount of organic iodine added, at 1.0 mg·kg^−1^, could have a good impact on energy, alkaline damage, intestinal mucosal growth, and the reproduction of beneficial microorganisms. In summary, when the level of organic iodine added is 0.5 mg·kg^−1^–1.0 mg·kg^−1^, it is beneficial for the promotion of the healthy development of the intestine.

Gut microbiota exert modulatory roles in metabolic pathways, immune processes, and neural functions [45], which have profound influences on the performance of health and production [46]. Studies have shown that an appropriately stable microbial ecosystem led to health conditions in rabbits [44,47]. The diversity and homeostasis of intestinal flora play a significant role in maintaining healthy development and reproductive performance of animals [48]. Yang Qing [49] investigated the effect of oral administration of a highly complex iodine solution on the intestinal flora of piglets. The results exhibited no significant difference in intestinal flora of piglets before and after administration. The coverage rate of bacteria in each group reached 99% through current experimental methods. Our results demonstrated that the Ace index, Shannon index, and Chao1 index of the experimental group were higher than those of the control group, but there was no significant difference. It could be seen that the abundance and diversity of microflora in the experimental group were higher than the control group, supposing that the addition of organic iodine tended to improve the composition of microbial community structure. The composition of intestinal microorganisms is complex and diverse; the main dominant bacteria are Firmicutes, Bacteroidetes, and Proteobacteria [50]. Our results are consistent with previous studies. According to LEfSe analysis, the abundance of Firmicutes in the experimental group increased and the abundance of Bacteroidetes decreased. With regard to the final weight of group III, it was significantly higher than the control group in the growth performance test of Fujian yellow rabbits. It could be suggested that the supplementation of organic iodine in the diets could increase the number of Firmicutes in the cecum of Fujian yellow rabbits and reduce the number of Bacteroidetes, thus changing the cecal flora structure of Fujian yellow rabbits and promoting the growth and development of experimental rabbits. Likewise, organic iodine in the diet can change the metabolic pathway factors of the cecal flora of Fujian yellow rabbits. There were some differences in the functional abundance of nutritional metabolism, genetic information processing, environmental information processing, and organic systems between the experimental group and the control group in terms of flora function prediction.

## 5. Conclusions

Diets supplemented with 1.5 mg∙kg^−1^ organic iodine had positive effects on the growth performance of Fujian yellow rabbits, as well as the humoral immune function. The addition of 0.5 mg∙kg^−1^ organic iodine in diets produced a respectable effect on the antioxidant capacity of the body and the immunoregulation effect by boosting the secretion of sIgA. Furthermore, adding organic iodine to the diet improved small intestine morphology, increased the activity of intestinal enzymes, maintained the dynamic balance of the intestinal environment to a certain extent, and also had the tendency to make the intestinal flora structure better, and promote the development of the intestine. In summary, the comprehensive effect was best when the amount of organic iodine was 0.5 mg∙kg^−1^. However, adding organic iodine has no obvious effect on improving the slaughtering performance of Fujian yellow rabbits, and will reduce muscle quality. Therefore, it would be interesting to pursue studies on the role and mechanism of dietary organic iodine levels on growth performance, intestinal development, and barrier function of rabbits.

## Figures and Tables

**Table 1:** Composition and nutrient levels of basal diet.

Components	(%)	Componsition ^2^	(%)
Corn	26.70	DE (MJ/kg)	10.46
Soybean meal	8.40	Crude protein	15.48
Wheat bran	15.40	Crude fiber	12.25
Alfalfa hay meal	28.50	Crude fat	3.63
Wheat middling	5.00	Calcium	0.73
Unified bran	6.00	Available phosphorus	0.37
Extruded soybean	5.00	Lysine	0.76
Methionine	0.10	Methionine+ cystine	0.45
CaHPO_4_	0.70		
Lysine	0.20		
Premix ^1^	4.00		
Total	100.00		

Note: ^1^ Provided the following per kilogram of diet: Cu 5 mg; Fe 100 mg; Zn 50 mg; Mn 30 mg; Mg 150 mg; I 1 mg (inorganic); Se 0.1 mg; VA 8000 IU; VD 800 IU; VE 50 mg; VB_1_ 2 mg; VB_2_ 6 mg; VB_6_ 2 mg; VB_12_ 0.01 mg; folic acid 1 mg; pantothenic acid 20 mg. ^2^ Nutrient contents were calculated values.

**Table 2:** Effects of organic iodine on the growth performance of Fujian yellow rabbits.

Items	Control	Group I	Group II	Group III
BW (kg)	2.02 ± 0.13 ^B^	2.11 ± 0.10 ^AB^	2.17 ± 0.06 ^AB^	2.31 ± 0.15 ^A^
ADG (g) (U/mg protein)	21.16 ± 1.82 ^B^	22.83 ± 1.51 ^AB^	23.85 ± 0.94 ^AB^	26.15 ± 2.14 ^A^
ADFI (g)	98.76 ± 0.75 ^B^	96.28 ± 1.27 ^C^	99.40 ± 1.09 ^B^	103.18 ± 0.87 ^A^
F/G (%)	4.68 ± 0.35 ^a^	4.23 ± 0.27 ^b^	4.17 ± 0.17 ^b^	3.96 ± 0.31 ^b^

In the same column, different lowercase letter superscripts mean notable differences (*p* < 0.05), while different capital letters show significant differences (*p* < 0.01). Values with the same or no letters mean no significant difference (*p* > 0.05).

**Table 3:** Effect of organic iodine on serum biochemical indexes of Fujian yellow rabbits.

Items	Control	Group I	Group II	Group III
T3 (ng/mL)	5.05 ± 0.52 ^B^	6.65 ± 0.46 ^A^	5.18 ± 0.51 ^B^	5.08 ± 0.70 ^B^
T4 (ng/mL)	115.15 ± 15.42 ^C^	203.30 ± 14.63 ^AB^	229.07 ± 14.42 ^A^	181.03 ± 13.66 ^B^
IgM (g/L) (U/mg protein)	0.42 ± 0.04 ^b^	0.43 ± 0.03 ^b^	0.43 ± 0.05 ^b^	0.65 ± 0.02 ^a^
IgA (g/L)	0.93 ± 0.09 ^c^	0.74 ± 0.19 ^c^	2.30 ± 0.31 ^a^	1.37 ± 0.27 ^b^
IgG (g/L)	24.94 ± 0.99 ^bc^	23.98 ± 1.71 ^c^	25.75 ± 1.77 ^b^	35.42 ± 2.24 ^a^

In the same column, different lowercase letter superscripts mean notable differences (*p* < 0.05), while different capital letters show significant differences (*p* < 0.01). Values with the same or no letters mean no significant difference (*p* > 0.05).

**Table 4:** Effect of organic iodine on intestinal mucosal immunity of Fujian yellow rabbits.

Items	Control	Group I	Group II	Group III
Duodena				
IL-1α (pg/mL)	26.74 ± 1.40 ^C^	44.76 ± 1.73 ^A^	37.36 ± 2.85 ^AB^	31.5 ± 5.05 ^BC^
IL-2 (pg/mL)	112.19 ± 8.90 ^C^	171.37 ± 6.86 ^A^	146.56 ± 15.68 ^AB^	128.18 ± 11.00 ^BC^
sIgA (μg/mL)	13.73 ± 1.37 ^C^	22.31 ± 2.53 ^A^	19.49 ± 0.48 ^AB^	16.27 ± 0.59 ^BC^
Jejunum				
IL-1α (pg/mL)	20.36 ± 1.11 ^C^	38.77 ± 1.66 ^A^	31.75 ± 2.94 ^AB^	24.95 ± 4.91 ^BC^
IL-2 (pg/mL)	91.11 ± 8.39 ^C^	151.04 ± 5.23 ^A^	126.53 ± 13.95 ^AB^	104.27 ± 11.65 ^BC^
sIgA (μg/mL)	13.73 ± 1.37 ^C^	19.12 ± 2.75 ^A^	15.81 ± 0.69 ^AB^	12.83 ± 0.62 ^BC^
Ileum				
IL-1α (pg/mL)	22.70 ± 3.81 ^B^	38.94 ± 4.52 ^A^	31.41 ± 3.90 ^AB^	26.40 ± 7.37 ^AB^
IL-2 (pg/mL)	92.01 ± 10.98 ^B^	158.07 ± 7.96 ^A^	119.21 ± 20.37 ^B^	115.18 ± 11.52 ^B^
sIgA (μg/mL)	12.09 ± 1.10 ^c^	18.01 ± 2.21 ^a^	16.16 ± 2.50 ^ab^	13. 66 ± 1.84 ^bc^

In the same column, different lowercase letter superscripts mean notable differences (*p* < 0.05), while different capital letters show significant differences (*p* < 0.01). Values with the same or no letters mean no significant difference (*p* > 0.05).

**Table 5:** Effect of organic iodine on gut morphometry and morphology of Fujian yellow rabbits.

Items	Control	Group I	Group II	Group III
Duodena				
Villous height (μm)	902.73 ± 139.83 ^ab^	915.98 ± 27.83 ^a^	778.85 ± 40.80 ^b^	912.68 ± 125.64 ^ab^
Crypt depth (μm)	115.23 ± 23.52 ^ab^	120.05 ± 27.28 ^a^	92.50 ± 11.62 ^b^	105. 8 ± 15.38 ^ab^
V/C	8.14 ± 2.15	7.90 ± 1.53	8.50 ± 0.89	8.93 ± 2.35
Goblet cells (NO./mm)	12.82 ± 4.09 ^b^	22.62 ± 12.74 ^ab^	18.69 ± 11.60 ^ab^	31.12 ± 14.57 ^a^
Jejunum				
Villous height (μm)	904.91 ± 69.8 ^ab^	814.01 ± 135.19 ^b^	913.89 ± 104. 99 ^ab^	991.77 ± 104.43 ^a^
Crypt depth (μm)	108.80 ± 18.61	109.31 ± 28.29	113.22 ± 19.01	108.25 ± 4.83
V/C	8.43 ± 0.91	7.86 ± 2.49	8.35 ± 2.27	9.18 ± 1.11
Goblet cells (NO./mm)	11.75 ± 6.29 ^B^	27.22 ± 10.28 ^AB^	42.97 ± 17.99 ^A^	26.31 ± 12.52 ^A^
Ileum				
Villous height (μm)	432.72 ± 75.59 ^ab^	415.98 ± 45.15 ^b^	469.62 ± 47.16 ^ab^	501.87 ± 48.96 ^a^
Crypt depth (μm)	110.81 ± 19.62 ^B^	161.93 ± 15.04 ^A^	116.61 ± 20.27 ^B^	93.91 ± 14.10 ^B^
V/C	4.00 ± 1.04 ^b^	2.59 ± 0.43 ^c^	4.13 ± 0.92 ^b^	5.41 ± 0.73 ^a^
Goblet cells (NO./mm)	13.89 ± 5.52 ^B^	9.59 ± 2.27 ^B^	24.62 ± 9.85 ^AB^	39.23 ± 15.99 ^A^

In the same column, different lowercase letter superscripts mean notable differences (*p* < 0.05), while different capital letters show significant differences (*p* < 0.01). Values with the same or no letters mean no significant difference (*p* > 0.05).

**Table 6:** Effect of organic iodine on the intestinal enzyme activity of Fujian yellow rabbits.

Items	Control	Group I	Group II	Group III
Amylase (µ/g)	7.94 ± 1.86 ^ab^	8.64 ± 1.66 ^a^	4.37 ± 2.85 ^b^	6.09 ± 1.90 ^ab^
Cellulase (µ/g)	206.51 ± 6.86 ^b^	281.35 ± 30.54 ^a^	247.02 ± 50.42 ^ab^	252.02 ± 13.75 ^ab^
Trypsin (µ/g)	15.89 ± 3.90 ^ab^	17.29 ± 3.59 ^a^	8.40 ± 6.20 ^b^	11.97 ± 4.18 ^ab^

In the same column, different lowercase letter superscripts mean notable differences (*p* < 0.05). Values with the same or no letters mean no significant difference (*p* > 0.05).

**Table 7:** Effect of organic iodine on cecal fermentation of Fujian yellow rabbits.

Items	Control	Group I	Group II	Group III
PH	6.75 ± 0.24 ^bc^	6.52 ± 0.31 ^c^	7.01 ± 0.21 ^ab^	7.12 ± 0.26 ^a^
NH_3_-N (mg/g)	2.99 ± 0.38 ^b^	4.70 ± 1.55 ^a^	3.23 ± 1.55 ^ab^	2.74 ± 0.22 ^b^
Acetic acid (μ/g)	1202.66 ± 345.23 ^bc^	1456.81 ± 184.86 ^ab^	1726.24 ± 152.92 ^a^	1007.56 ± 37.30 ^c^
Propionic acid (μ/g)	180.23 ± 35.42 ^a^	211.95 ± 32.23 ^a^	220.88 ± 48.67 ^a^	108.74 ± 31.83 ^b^
Butyric acid (μ/g)	128.43 ± 46.61	177.68 ± 105.55	113.34 ± 14.85	75.14 ± 20.13

In the same column, different lowercase letter superscripts mean notable differences (*p* < 0.05). Values with the same or no letters mean no significant difference (*p* > 0.05).

**Table 8:** Effect of organic iodine on cecal microbial alpha diversity of Fujian yellow rabbits.

Items	Control	Group I	Group II	Group III
Shannon	7.15 ± 0.31	7.60 ± 0.33	7.76 ± 0.57	7.84 ± 0.18
Simpson	0.975	0.978	0.982	0.986
Ace	1222.01 ± 6.02	1368.89 ± 76.50	1275.89 ± 84.37	1403.04 ± 76.16
Chao1	1212.32 ± 12.53	1354.28 ± 76.16	1274.16 ± 92. 82	1393.19 ± 373.37
Coverage	0.998	0.997	0.998	0.997

## Data Availability

Data are contained within the article.
